# A novel mitochondrial genome architecture in thrips (Insecta: Thysanoptera): extreme size asymmetry among chromosomes and possible recent control region duplication

**DOI:** 10.1186/s12864-015-1672-4

**Published:** 2015-06-09

**Authors:** Aaron M. Dickey, Vivek Kumar, J. Kent Morgan, Antonella Jara-Cavieres, Robert G. Shatters, Cindy L. McKenzie, Lance S. Osborne

**Affiliations:** Subtropical Insects Research Unit, US Horticultural Research Laboratory, Fort Pierce, USA; Mid-Florida Research & Education Center, University of Florida, Apopka, USA; Present Address: Agricultural Research Service, US Department of Agriculture, Clay Center, USA; Present Address: J. Kent Morgan Consulting, Fort Pierce, USA; Indian River Research and Education Center, University of Florida, Fort Pierce, USA; US Department of Agriculture, Agricultural Research Service, Fort Pierce, USA

## Abstract

**Background:**

Multipartite mitochondrial genomes are very rare in animals but have been found previously in two insect orders with highly rearranged genomes, the Phthiraptera (parasitic lice), and the Psocoptera (booklice/barklice).

**Results:**

We provide the first report of a multipartite mitochondrial genome architecture in a third order with highly rearranged genomes: Thysanoptera (thrips). We sequenced the complete mitochondrial genomes of two divergent members of the *Scirtothrips dorsalis* cryptic species complex. The East Asia 1 species has the single circular chromosome common to animals while the South Asia 1 species has a genome consisting of two circular chromosomes. The fragmented South Asia 1 genome exhibits extreme chromosome size asymmetry with the majority of genes on the large, 14.28 kb, chromosome and only *nad6* and *trnC* on the 0.92 kb mini-circle chromosome. This genome also features paralogous control regions with high similarity suggesting a very recent origin of the *nad6* mini-circle chromosome in the South Asia 1 cryptic species.

**Conclusions:**

Thysanoptera, along with the other minor paraenopteran insect orders should be considered models for rapid mitochondrial genome evolution, including fragmentation. Continued use of these models will facilitate a greater understanding of recombination and other mitochondrial genome evolutionary processes across eukaryotes.

**Electronic supplementary material:**

The online version of this article (doi:10.1186/s12864-015-1672-4) contains supplementary material, which is available to authorized users.

## Background

Multipartite mitochondrial genomes are common among plants [1, 2], but are quite rare among animals, the majority of which possess a single, 10–35 kb circular chromosome [3]. Among insects, Phthiraptera [4, 5] and Psocoptera [6] are the only two insect orders containing species with multipartite mitochondrial genomes. These orders, together with the Hemiptera and Thysanoptera, comprise the insect superorder Paraneoptera.

Thysanoptera contains ~5500 species of tiny (very often <1 mm), linear insects commonly known as thrips. The size and shape of thrips helps explain their significant representation on many invasive species lists and several species in this order are globally important crop pests [7]. *Scirtothrips dorsalis* (Hood) is comprised of multiple cryptic species, two of which have established in the continental USA since 2005 [8] and 2011 [9]. These species originate from different parts of the world, have different climatic requirements and differ in host preference so discriminating between them at the molecular level is a high priority [9]. Toward this end, the complete mitochondrial genomes of both recently adventive species, South Asia 1 (SA1) and East Asia 1 (EA1), were sequenced for comparative genomic analysis. This was done initially using next-generation sequencing (NGS) data, and subsequently validated using Sanger sequencing. From these experiments, it was concluded that EA1 has a traditional single chromosome mitochondrial genome, while SA1 has a fragmented genome consisting of two chromosomes.

## Results

**Fig. 1:**
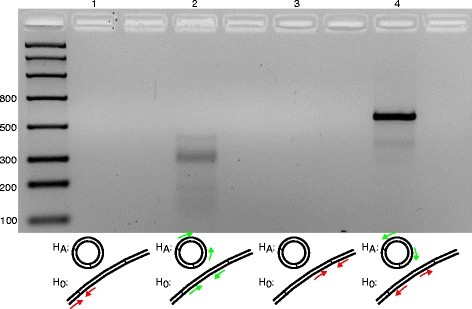
Confirming the mini-circle chromosome in *Scirtothrips dorsalis* cryptic species SA1. Specific primers spanning putative *nad6*/*nad4* and *nad6*/*rrnL* gene boundaries fail to amplify (H_0_, lanes 1 and 3 respectively). Internal primers amplify a portion of *nad6* (lane 2) while the reverse compliment of these primers (H_A_, lane 4) completes the 921 bp *nad6* chromosome. Non-numbered lanes are negative controls for each PCR

**Fig. 2:**
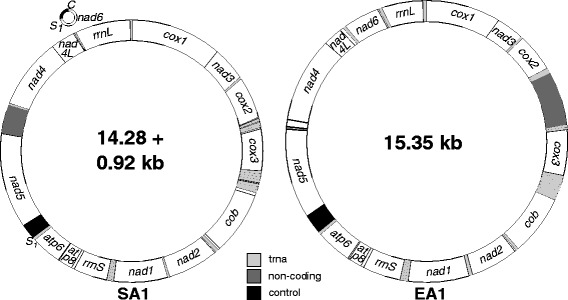
The *Scirtothrips dorsalis* South Asia 1 cryptic species (SA 1) is the first thysanopteran discovered to possess a bipartite mitochondrial genome. The genome is consists of two chromosomes. The *S. dorsalis* East Asia 1 cryptic species (EA 1) has the single circular chromosome common to animals

BLAST queries of NGS contigs identified all mitochondrial protein coding and ribosomal genes in both cryptic species except *nad4L* and *atp8*. Subsequent scaffolding of the NGS contigs of provisional mitochondrial origin yielded a completed circular mitochondrial chromosome for EA1, but we were unable to circularize the SA1 genome using NGS data and instead inferred a single large chromosome by placing two short gap fillers consisting of EA1 sequence (lengths 20 and 37 bp) into the inferred single SA1 chromosome prior to capillary sequencing validation. These fillers were on either side of a complete *nad6* gene sequence. Attempting to resolve the missing sequence in the SA1 genome surrounding the *nad6* gene yielded an unexpected result. Primers designed to span these gaps across the putative *nad6*/*nad4* and *nad6*/*rrnL* gene boundaries failed (Fig. 1, lanes 1 and 3, null hypothesis rejected), while the reverse complement of internal *nad6* primers produced a PCR product (Fig. 1, lane 4, alternative hypothesis validated). The lane 4 PCR reaction should only have yielded a product if the genome was fragmented with *nad6* on a second mini-loop chromosome. Furthermore, the sequence of this product was mitochondrial in origin, contained a control region (also called the A + T-rich region) and also a *trnS*_*1*_ gene (anticodon tct with d-loop). These paralogs were highly similar (98.35 % and 100 % respectively) to those in the larger, but *nad6* and *trnC* deficient, mitochondrial chromosome. This experiment simultaneously validated the existence of a second non-redundant mitochondrial chromosome in the SA1 cryptic species of *S. dorsalis* (Fig. 2) and confirmed the presence of all 37 mitochondrial genes in the SA1 genome. Furthermore, the complete intervening sequence between *nad4* and *rrnL* in the large SA1 chromosome was validated using capillary sequencing primers (primer pairs 5–8 on page 2 of Additional file 1) and it was confirmed that no duplicate *nad6* gene was present. This is the location of *nad6* in all other thysanoptera including EA1. In total, the mitochondrial genomes were validated using capillary sequencing covering 99.95 % of the SA1 genome and 74.48 % of the EA1 genome.

Representations of the final genomes appear in Fig. 2 and all 37 mitochondrial genes common to insects are present in both species (Table 1). The SA1 mitochondrial genome consists of 2 chromosomes: a mini-loop designated the *nad6* chromosome or SA1 Chromosome 2, and a larger chromosome designated SA1 Chromosome 1. SA1 Chromosome 2 is 921 bp in length, contains non-redundant *nad6* and *trnC* genes and also *trnS1* and the control region, presumably duplicated from Chromosome 1. The 14,283 bp SA1 Chromosome 1 contains all expected mitochondrial genes except *nad6* and *trnC*. Sequences for SA1 Chromosome 1 and 2 have been deposited in GenBank with accession numbers KM349827 and KM349828. The single EA1 chromosome is 15,343 bp long and has been assigned the accession number KM349826. The A + T content of the two SA1 chromosomes are 77.12 % (Chromosome 1) and 81.54 % (Chromosome 2). The A + T content of the two genomes are 77.39 % (SA1) and 75.74 % (EA1).

**Table 1:** Gene order and sequence difference among *Scirtothrips dorsalis* South Asia 1 (SA1) and East Asia 1 (EA1) cryptic species

SA1	Strand^a^	EA1	Strand^a^	Sequence difference^b^
*cox1*	J	*cox1*	J	11.76 %
*nad3*	J	*nad3*	J	19.21 %
*L*_*2*_	J	*L*_*2*_	J	5.97 %
*cox2*	J	*cox2*	J	12.83 %
*D*	J	*D*	J	13.43 %
*R*	J	*R*	J	1.47 %
	J	Non-coding	J	Non-homologous
*G*	J	*G*	J	9.23 %
*K*	J	*K*	J	4.76 %
*cox3*	J	*cox3*	J	11.28 %
*I*	J	*I*	J	14.29 %
*L*_*1*_	J	*L*_*1*_	J	9.23 %
*T*	J	*T*	J	7.81 %
*P*	J	*P*	J	9.23 %
*N*	J	*N*	J	10.17 %
*E*	J	*E*	J	12.12 %
*Q*	J	*Q*	J	2.94 %
*cob*	J	*cob*	J	13.51 %
*Y*	J	*Y*	N	10.61 %
*nad2*	J	*nad2*	J	19.64 %
*W*	J	*W*	J	7.35 %
*nad1*	J	*nad1*	J	17.80 %
*M*	J	*M*	J	6.25 %
*A*	J	*A*	J	3.03 %
*F*	N	*F*	J	4.41 %
*rrnS*	J	*rrnS*	J	12.42 %
*atp8*	J	*atp8*	J	21.14 %
*atp6*	J	*atp6*	J	18.82 %
*S*_*1*_	J	*S*_*1*_	J	11.67 %
Control	J	Control	J	29.15 %
*nad5*	N	*nad5*	N	16.34 %
Non-coding	N		N	Non-homologous
*H*	N	*H*	N	15.15 %
*nad4*	N	*nad4*	N	19.12 %
*nad4L*	N	*nad4L*	N	20.63 %
***C***	**J**	*C*	J	8.06 %
***nad6***	**J**	*nad6*	J	21.37 %
*V*	J	*V*	J	13.33 %
*rrnL*	J	*rrnL*	J	12.09 %
*S*_*2*_	J	*S*_*2*_	J	4.41 %

Genomes are linearized and SA1 Chromosome 2 (bold) is inserted into its proposed ancestral location; tRNA genes are abbreviated to the letter of their coding amino acid, *S*_*1*_ (anticodon tct), *S*_*2*_ (tga), *L*_*1*_ (tag), *L*_*2*_ (taa)

^a^Majority (J), Minority (N) coding strands

^b^Indels were treated as a single difference regardless of size and non-overlapping regions at the termini of tRNA alignments were ignored

**Fig. 3:**
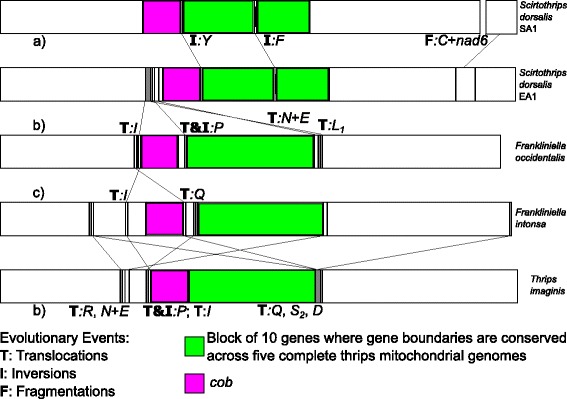
Rates of various evolutionary events inferred from five linearized complete thrips mitochondrial genomes [current study, 13–15]. **a** Inversions and a fragmentation event are present among members of a single cryptic species complex. **c** Translocations are more common among morphologically different members of a genera, and **b** increasingly common among different genera

Both the order and the coding strand of protein coding and ribosomal RNA genes are identical to the other three thrips species with complete mitochondrial genomes available: *Thrips imaginis* [GenBank:NC_004371], *Frankliniella occidentalis* [GenBank:JN835456] and *Frankliniella intonsa* [GenBank:JQ917403]. Translocations and inversions between *S. dorsalis* and the other thrips species are relegated to tRNA genes and much of this activity either precedes or follows *cob* (Fig. 3). The SA1 genome has *trnF* coded on the minority strand (the strand encoding the fewest genes and designated N) while the EA1 genome has *trnY* coded on the minority strand. Aside from these two inversions, the order and strand location of all genes is the same (Table 1). In addition, the EA1 and SA1 genomes contain long (623 and 242 bp respectively) non-coding regions with no homolog in the other species (Fig. 2). These lack the requisite features of control regions such as the T-stretch and A + T-rich segments. They also occur in different locations of the two genomes, between *trnR* and *trnG* in EA1 and between *nad5* and *trnH* in chromosome 1 of SA1 (Table 1). The two genomes differ by 15.17 % across all 38 homologs (37 genes plus the control region) and by 16.22 % across the 13 protein coding genes.

There were 15 changes between the NGS contig containing the SA1 *nad6* gene to the completed SA1 Chromosome 2 validated by capillary sequencing. This corresponds to an error rate in NGS of about 1.6 %. Seven of these changes occurred within the first or last 80 nucleotides of the NGS contig, eleven occurred in A or T stretches ≥8 nt, while three changes did not meet either of these criteria.

## Discussion

The complete mitochondrial genomes were sequenced from two members of the *S. dorsalis* species complex using next-generation DNA sequencing and validated using capillary sequencing. We found that the two genomes are highly divergent facilitating easy discrimination. Levels of divergence were comparable to other crop pest cryptic species complexes [10] and 5–20 fold higher than among cryptic species of mosquitoes [11]. Moreover, an important discovery emerged when comparing the two genomes. One species has the single circular mitochondrial chromosome common to animals while the other has a fragmented genome consisting of one large chromosome and one small chromosome. The event giving rise to this architecture probably occurred quite recently based on high similarity among paralogs and the very small size of SA1 Chromosome 2. The complete control region and *trnS*_*1*_, appear to have been duplicated either preceding or during this fragmentation event. These paralogs show little divergence among chromosomes (1.65 % and 0 % for the control region and *trnS*_*1*_ respectively). In the case of the human body louse, paralogous control regions exhibited higher divergences ranging from 3 to 23 % [5] suggesting this species has been multipartite for a longer period of time than has the SA1 cryptic species of *Scirtothrips dorsalis*.

Several additional putative tRNA genes were found however none appear to have homologs in the other cryptic species as such are either pseudogenes or were misidentified by the search algorithm. ARWEN is known to be a highly sensitive search algorithm with a corresponding high false positive rate [12]. Given that the two *trnS*_*1*_ paralogs within the SA1 genome are identical, they should both be functional though the duplicated *trnS*_*1*_ on SA1 Chromosome 2 is redundant.

Methodologically, the bipartite nature of the mitochondrial genome of the South Asia 1 cryptic species was confirmed using a PCR experiment whereby the null hypothesis of a single chromosome was rejected and the alternative hypothesis of a separate mini-circle chromosome containing *nad6* was accepted (Fig. 1). The absence of *nad6* from the large chromosome of this species was also confirmed by sequencing the entire region between *nad4* and *rrnL* where *nad6* normally occurs in thrips (primer pairs 5–8 on page 2 of Additional file 1). This provided additional evidence that *nad6* is not present in this intervening region on the large SA1 chromosome, is not duplicated in the genome and is non-redundant. Furthermore, given the apparent variation in PCR product brightness seen in Fig. 1, we sought to eliminate the possibility that reduced efficiency primer pairs might have overlooked coexistence among unipartite and bipartite genomes in SA1. The apparently higher efficiency primers (lane 4 in Fig. 1; primer pair 16 on page 2 of Additional file 1) were also used in the failed PCR reactions (lanes 1 and 3 in Fig. 1; primer pairs 13 and 15 on page 2 of Additional file 1). To confirm this result, three additional PCR experiments were performed for each putative gene boundary which would be found in a coexistence scenario using different primers (pairs 17–22 on page 2 of Additional file 1). All these experiments failed to yield a product increasing our confidence in the interpretation of an exclusively bipartite genome in SA1. 99.95 % of the complete genome of the SA 1 cryptic species has been Sanger validated and both chromosomes have been successfully circularized. In the EA 1 cryptic species, the single chromosome genome was successfully circularized using only next-generation sequencing data and, in addition, has been ~75 % Sanger validated.

With the exception of the fragmentation event giving rise to SA1 Chromosome 2, all gene boundaries are shared among cryptic species within the *S. dorsalis* complex, with two tRNA inversions (Fig. 3, Table 1). In addition, the location of protein coding and ribosomal RNA genes is constant among all five complete thrips mitochondrial genomes [current study, 13–15] with all differences accounted for by tRNA gene translocations or inversions (Fig. 3). Given that five complete thrips mitochondrial genomes have been sequenced representing both transgeneric, congeneric, and cryptic species pairwise comparisons, some initial predictions can be made about the relative rates of different types of evolutionary events in animals with highly rearranged mitochondrial genomes. First, tRNA gene inversions may be the most common event given they have occurred among lineages representing members of the same cryptic species complex (Fig. 3a). Second, tRNA gene translocations have occurred more frequently in the lineages leading to morphologically distinguishable congeners and even more frequently among lineages representing different genera (Fig. 3b-c). Shifts to a multipartite architecture within a genus may also be common (Fig. 3a), but testing this will require discovery of additional examples. All five complete thrips mitochondrial genomes share a block of 10 genes (*trnY* to *atp6*) where all gene boundaries are shared (Fig. 3). The five genomes represent three genera, all in the pestiferous subfamily Thripinae. Additional complete genomes are needed from thrips in different subfamilies and suborders to make better inferences about these patterns and their phylogenetic significance across Thysanoptera.

At 921 bp, SA1 chromosome 2 is the smallest mitochondrial chromosome documented in insects. It is about 1/3 the size of the smallest mini-circle chromosome in the human body louse [5] and about 45 % the size of the smallest mini-circle chromosome in the louse *Damalinia* [4]. *Scirtothrips dorsalis* lacks multiple control regions found in other thrips with the exception of the paralog in SA1 chromosome 2 (Fig. 2). The small size of the SA1 mini-circle chromosome is likely a consequence of its recent evolution and selection may be for more expansive control regions and concomitant increased size of mini-circle chromosomes with the progression of time as in lice [5].

Thrips in the genus *Frankliniella* have multiple control regions in their mitochondrial genomes [13, 14]. In contrast, EA1 and SA1 Chromosome 1 contain relatively large non-coding regions with no obvious control region motifs and no obvious homology among cryptic species. These two sequences also occur in widely separated parts of their respective chromosomes (Table 1). While multiple control regions are not present in individual mitochondrial chromosomes for *S. dorsalis* cryptic species, their presence in *Frankliniella* raises a possible connection between multiple control regions and a fragmented architecture. For example, if multi-partism can be reversed, it might lead to multiple control regions in a single chromosome. Alternatively, duplicated control regions in a single mitochondrial chromosome may precede and help facilitate the evolution of multi-partism.

The discovery of a mini-circle chromosome in Thysanoptera was assisted by large scale genomic data from next-generation sequencing. Interestingly, large-scale genomic data was also needed to discover the fragmented mitochondrial genome architecture in the human body louse [5]. This indicates an important role for NGS platforms in mitochondrial evolution research. For this type of study, 1.6 % should be considered a high estimate for the true error rate between NGS and capillary sequencing. This estimated error rate is likely exaggerated by 1) the small chromosome size used for calculation, 2) the AT rich nature of these data and 3) higher error rates at the ends of contigs due to mis-assignment of raw reads. The issue of small chromosome size is peculiar to this study while the issue of uncertain lengths of poly A’s and poly T’s is a known source of error in Ion Torrent data and this type of error is likely to be elevated in AT rich taxa. In addition, an elevated error rate at the ends of contigs has been documented in other NGS studies [16] and is probably a function of the trade-off between contig length and quality during assembly of NGS raw reads. If error due to poly A or T stretches and the ends of contigs are ignored, the error rate for the SA1 mini-circle chromosome between NGS and Sanger sequencing is reduced from 1.6 % to 0.3 %. Despite these challenges, NGS provides a powerful tool for *de novo* mitochondrial genome construction. In hindsight, our inability to complete the expected single mitochondrial chromosome for the SA1 cryptic species of *S. dorsalis* using NGS data was the first indication that the genome could be fragmented.

## Conclusions

This is the first report of a bipartite mitochondrial genome architecture in the insect order Thysanoptera and the latest development in the study of multipartite mitochondrial genomes in bilateria, an emerging area of inquiry with high potential to revolutionize our overall understanding of mitochondrial genome evolution. Among insects, this phenomenon has only been found in the three minor paraneopteran orders, Phthiraptera, Psocoptera, and Thysanoptera. The phenomenon has not yet been reported in the major paraneopteran order, Hemiptera, despite 69 complete mitochondrial genomes [17]. The three orders with multipartite members also show the highest degree of genome rearrangement [6, 14, 18] suggesting the two phenomena may be correlated. All three minor orders should be considered as animal models for rapid mitochondrial genome evolution, including genome fragmentation. We believe that continued development of these models will ultimately facilitate high resolution in vitro testing of mitochondrial recombination e.g. [19] and a greater understanding of mitochondrial evolution across eukaryotes.

## Methods

Next-generation sequencing (NGS) using the Ion Torrent PGM system was conducted on two samples of *S. dorsalis. Scirtothrips dorsalis* is a complex comprised of multiple cryptic and non-cryptic species [9] and the two samples for NGS were from the phylogenetic extremities within this complex. DNA extraction details for the East Asia 1 cryptic species (EA1) and the South Asia 1 cryptic species (SA1) can be found in [9] and [20] respectively; importantly the SA1 sample contained 97 individuals from an invasive population in Florida, USA. The Ion Torrent system (Thermo Fisher, Waltham, MA) was used for next-generation sequencing and details were consistent across samples including reagent kits, hardware, and preliminary analyses to create contigs from raw sequencing reads. These details can be found in [20].

Contigs with likely mitochondrial origin were identified using BLAST [21] comparison to the existing Thysanoptera mitochondrial genomes [13–15] and scaffolds were created from identified contigs to produce putative complete genomes. Primers were designed using Primer 3 [22] to amplify both genomes and validate with traditional capillary sequencing (Additional file 1). Polymerase chain reactions were run using GoTaq (Promega, Madison, WI) kits, PCR products were visualized on a 1.5 % agarose gel via electrophoresis, cleaned up using Nucleospin (Machery-Nagel, Bethlehem, PA) clean-up kits, and directly sequenced bidirectionally using a BigDye Terminator cycle sequencing kit and an 3730XL DNA sequencer (both Thermo Fisher). To maximize the template available for capillary sequencing validation, whole genome amplification was done using the GenomiPhi V2 kit (GE Healthcare, Piscataway, NJ). Sequences were contiged, traces inspected, and base calls edited using Sequencher v4.8 software (Gene Codes, Ann Arbor, MI). Sequencher was also used to align homologs and paralogs. Sequences were compared to the genomes developed with the NGS data. Open reading frames were identified through BLAST comparison to existing thrips mitochondrial genomes for protein coding and ribosomal RNA genes. Capillary sequence validation was incomplete for EA1 due to exhausting the DNA template. Thus, a minimal number of manual edits to the EA1 genome were made to obtain ORF’s with similar amino acid sequences among cryptic species. Transfer RNA genes were identified using ARWEN [12]. The bipartite nature of the South Asia 1 mitochondrial genome was confirmed by designing primers that were the reverse compliment of internal *nad6* gene primers (Additional file 1). No human subjects were used in this study.

### Availability of supporting data

The data supporting the results of this article are available in the GenBank repository.

EA1 Genome, KM349826, http://www.ncbi.nlm.nih.gov/nuccore/KM349826.1.

SA1 Chromosome 1, KM349827, http://www.ncbi.nlm.nih.gov/nuccore/KM349827.1.

SA1 Chromosome 2, KM349828, http://www.ncbi.nlm.nih.gov/nuccore/KM349828.1.

## Additional file

Additional file 1:
**Capillary sequencing primer pairs used to validate the NGS data.**

## References

[1] Palmer JD, Herbo LA (1987). Unicircular structure of the Brassica hirta mitochondrial genome. Curr Genet.

[2] Sugiyama Y, Watase Y, Nagase M, Makita N, Yagura S, Hirai A, Sugiura M (2005). The complete nucleotide sequence and multipartite organization of the tobacco mitochondrial genome: comparative analysis of mitochondrial genomes in higher plants. Mol Genet Genomics.

[3] Lupi R, de Meo PD, Picardi E, D'Antonio M, Paoletti D, Castrignanò T, Pesole G, Gissi C (2010). MitoZoa: a curated mitochondrial genome database of metazoans for comparative genomics studies. Mitochondrion.

[4] Cameron SL, Yoshizawa K, Mizukoshi A, Whiting MF, Johnson KP (2011). Mitochondrial genome deletions and minicircles are common in lice (Insecta: Phthiraptera). BMC Genomics.

[5] Shao R, Kirkness EF, Barker SC (2009). The single mitochondrial chromosome typical of animals has evolved into 18 minichromosomes in the human body louse Pediculus humanus. Genome Res.

[6] Wei D-D, Shao R, Yuan M-L, Dou W, Barker SC, Wang J-J (2012). The multipartite mitochondrial genome of Liposcelis bostrychophila: insights into the evolution of mitochondrial genomes in bilateral animals. PLoS One.

[7] Morse JG, Hoddle MS (2006). Invasion biology of thrips. Annu Rev Entomol.

[8] Kumar V, Kakkar G, McKenzie CL, Seal DR, Osborne LS, Soloneski S, Larramendy M (2013). An overview of Chilli Thrips, Scirtothrips dorsalis (Thysanoptera: Thripidae) biology, distribution and management. Weed and Pest Control - Conventional and New Challenges.

[9] Dickey AM, Kumar V, Hoddle MS, Funderburk JE, Morgan JK, Jara-Cavieres A, Shatters Jr RG, Osborne LS, McKenzie CL. The Scirtothrips dorsalis species complex: endemism and invasion in a global pest. PLoS One. 2015;10(4), e0123747. 10.1371/journal.pone.0123747 PMC4404325 25893251

[10] Wang H-L, Yang J, Boykin LM, Zhao Q-Y, Li Q, Wang X-W, Liu S-S (2013). The characteristics and expression profiles of the mitochondrial genome for the Mediterranean species of the Bemisia tabaci complex. BMC Genomics.

[11] Krzywinski J, Li C, Morris M, Conn JE, Lima JB, Povoa MM, Wilkerson RC (2011). Analysis of the evolutionary forces shaping mitochondrial genomes of a neotropical malaria vector complex. Mol Phylogenet Evol.

[12] Laslett D, Canbäck B (2008). ARWEN: a program to detect tRNA genes in metazoan mitochondrial nucleotide sequences. Bioinformatics.

[13] Yan D, Tang Y, Hu M, Liu F, Zhang D, Fan J (2014). The mitochondrial genome of Frankliniella intonsa: insights into the evolution of mitochondrial genomes at lower taxonomic levels in Thysanoptera. Genomics.

[14] Yan D, Tang Y, Xue X, Wang M, Liu F, Fan J (2012). The complete mitochondrial genome sequence of the western flower thrips Frankliniella occidentalis (Thysanoptera: Thripidae) contains triplicate putative control regions. Gene.

[15] Shao R, Barker SC (2003). The highly rearranged mitochondrial genome of the plague thrips Thrips imaginis (Insecta: Thysanoptera): convergence of two novel gene boundaries and an extraordinary arrangement of rRNA genes. Mol Biol Evol.

[16] Chin CS, Alexander DH, Marks P, Klammer AA, Drake J, Heiner C, Clum A, Copeland A, Huddleston J, Eichler EE, Turner SW, Korlach J (2013). Nonhybrid, finished microbial genome assemblies from long-read SMRT sequencing data. Nat Methods.

[17] Cameron SL (2014). Insect mitochondrial genomics: implications for evolution and phylogeny. Annu Rev Entomol.

[18] Covacin C, Shao R, Cameron S, Barker S (2006). Extraordinary number of gene rearrangements in the mitochondrial genomes of lice (Phthiraptera: Insecta). Insect Mol Biol.

[19] Shao R, Barker SC (2011). Chimeric mitochondrial minichromosomes of the human body louse, Pediculus humanus: evidence for homologous and non-homologous recombination. Gene.

[20] Dickey AM, Trease AJ, Jara-Cavieres A, Kumar V, Christenson MK, Potluri L-P, Morgan JK, Shatters Jr RG, McKenzie CL, Davis PH. Estimating bacterial diversity in Scirtothrips dorsalis Hood (Thysanoptera: Thripidae) via next generation sequencing. Fla Entomol. 2014;92(1):362–6. 10.1653/024.097.0204 PMC4222051 25382863

[21] Camacho C, Colulouris G, Avagyan V, Ma N, Papadopoulos J, Bealer K, Madden T (2009). BLAST+: architecture and applications. BMC Bioinforma.

[22] Rozen S, Skaletsky H (2000). Primer3 on the WWW for general users and for biologist programmers. Methods Mol Biol.

