# Supplementary material for: A novel mitochondrial genome architecture in thrips (Insecta: Thysanoptera): extreme size asymmetry among chromosomes and possible recent control region duplication
Source: BMC Genomics. 2015 Jun 9;16(1):439. doi: 10.1186/s12864-015-1672-4 (PMC4460840; doi:10.1186/s12864-015-1672-4)
Supplement: Additional file 1: — Capillary sequencing primer pairs used to validate the NGS data. [file 12864_2015_1672_MOESM1_ESM.pdf]

Additional File: Capillary sequencing primers, estimated fragment size, and annealing temperature (T_A) used to validate next generation sequenced *Scirtothrips dorsalis* mitochondrial genomes.

Primer[a]	Sequence	Size	T_A
SD_SA1_c1_14063J	GCTACCTCGATGTTGAATTAAGG	969	55
SD_SA1_c1_707N	AAACTTCTGGGTGTCCAAAAA		
SD_SA1_c1_672J	TTTTTGATCCAGGAGGAGGA	522	55
SD_SA1_c1_1174N	CACCTGCAAAAATAGCAAAAA		
SD_SA1_c1_1063J	TTTTACTCTAGGAGGTTTAACAGGAG	926	45
SD_SA1_c1_1940N	AAAACGATGTCAATACTTTAGGAG		
SD_SA1_c1_1947J	TCAAAAATGAGATTTATTCTCCTAAA	805	51
SD_SA1_c1_2704N	GGTCGAAACTAAGAACAGAAGCA		
SD_SA1_c1_2045J	TCATAGAAGAATTCCACGATTACAC	705	50
SD_SA1_c1_2704N	GGTCGAAACTAAGAACAGAAGCA		
SD_SA1_c1_2597J	AATTTGTGGAACAGGACATGC	985	55
SD_SA1_c1_3539N	TGAATTCCATGAAATCCTGTAGC		
SD_SA1_c1_3119J	TGCATGAAGATGATGACGTG	562	54
SD_SA1_c1_3642N	TCATGCTGCTGCTTCAAATC		
SD_SA1_c1_3119J	GCAGATTCAGTCTATGGGTCAAC	949	50
SD_SA1_c1_4480N	CCCCATTAGCATGTAAAATTCG		
SD_SA1_c1_4108J	ATTTTCAGTATAAAGAAGATAATGT	273	45
SD_SA1_c1_4332N	TCCTAAAATTGATCCAAAATTTCAC		
SD_SA1_c1_4446J	TCAATAGCATTTCAAAGAGTTATTCAT	583	55
SD_SA1_c1_4979N	CCGGATCTCCTAAATAATATGGAA		
SD_SA1_c1_4920J	CCTTTTAAGCCATATTTTGTTTCG	911	40
SD_SA1_c1_5780N	TTTCAATTAAAGAAATAACTCAAAACTG		
SD_SA1_c1_5661J	TTTTCTTATTCAAAGAGTTTCTTC	956	51
SD_SA1_c1_6567N	TCGTTCTAAAAGGGTAATAAAAGC		
SD_SA1_c1_5950J	TTGCATCTATTAGAGGTTTAACT	548	47
SD_SA1_c1_6451N	TTGAAAATTAATAGTTTGCTTTAAC		
SD_SA1_c1_6209J	CGGGGATCCCACCTTTTT	556	50
SD_SA1_c1_6729N	CTCAAGGGTGGGCAAATAAA		
SD_SA1_c1_6406J	AAAATAGGTTTCCATGATTTCTC	943	55
SD_SA1_c1_7306N	GAGGTAATGTAGCTCGGATTC		
SD_SA1_c1_7126J	TGATTTCGCAGAAGGAGAAAG	988	47
SD_SA1_c1_8067N	TTTCCTTAAAACGGGGATATAGAAC		
SD_SA1_c1_7967J	TTTTCCCAGAGTAAAAACAGAAGA	148	54
SD_SA1_c1_8067N	TTTCCTTAAAACGGGGATATAGAAC		
SD_SA1_c1_8016J	AAATTTTAGGGATGCTTG	932	55
SD_SA1_c1_8908N	ATTAAAGCTGTTGGTGTTCC		
SD_SA1_c1_8294J	ACAAATTGCCCGTCACTCTC	998	55
SD_SA1_c1_9252N	TATACGCGCGTGGTTTAACA		
SD_SA1_c1_9041J	CCTTCTTTTAACACTTTTGGGAAA	254	55
SD_SA1_c1_9252N	TATACGCGCGTGGTTTAACA		
SD_SA1_c1_9189J	TTCAGATGAATCCGATTACACA	595	53
SD_SA1_c1_9728N	TGTTTGTTTATTAAGGTTATCAATATGC		
SD_SA1_c1_9275J	AAACCACGCGCGTATAAAAC	213	52
SD_SA1_c1_9446N	TTTTTAATTTTGCCTTAATTTGC		
SD_SA1_c1_9597J	TTCAATGTATCCTACTGAGACTTTCG	950	55
SD_SA1_c1_10497N	AGCAATAGCTGCTCCTACACC		
SD_SA1_c1_10248J	CCAGCATGAGTAACAAGGTGAA	989	55
SD_SA1_c1_11194N	TCGCAGAATTAAAGCAACAAAA		

[a]Primer naming convention: species complex_cryptic species_chromosome (cryptic species SA1 only)_location in genome and majority coding (J) or minority coding (N)

Additional file continued: Capillary sequencing primers, estimated fragment size, and annealing temperature (T_A) used to validate next generation sequenced *Scirtothrips dorsalis* mitochondrial genomes.

Primer[a]	Sequence	Size	T_A	Note[b]
SD_SA1_c1_10464J	TCGAATTATAATATATACACCTGCAGTAA	736	53	
SD_SA1_c1_11390N	AAAAACGTTGATTTGTGGAATC			
SD_SA1_c1_10975J	GAGAAGAAATATAATCCAAATATAAAGG	949	40	
SD_SA1_c1_12116N	TCATGTAGAGGCTCCGGTTT			
SD_SA1_c1_11217J	TTGTTGCTTTAATTCTGCGACT	318	54	
SD_SA1_c1_11489N	TCCTGTTATTTATCCGAATTTTTCA			
SD_SA1_c1_11217J	TTGTTGCTTTAATTCTGCGACT	455	54	
SD_SA1_c1_11626N	TTTGTTCTTCGTTTTCTTGAAGATT			
SD_SA1_c1_12009J	CCCCCTCAAATTCTGATAAAA	1256	51	Pairs span *nad4* and *rrnL* in SA1
SD_SA1_c1_13219N	TCATGATACACAAGGAACAAAA			
SD_SA1_c1_12940J	CAAGCCTCCAAAAGCAAAAG	964	55	
SD_SA1_c1_13302N	TTAACAATTATGAAACATTGAACAC			
SD_SA1_c1_12378J	TTTCCCCAGCCTAAAATAATG	428	53	
SD_SA1_c1_12765N	TTTGGTTCTGGGAATTTTCG			
SD_SA1_c1_12673J	GGACAGATAGCTATTAGACCCAACA	288	55	
SD_SA1_c1_12917N	TTTGGTTCTGGGAATTTTCG			
SD_SA1_c1_13241J	AAAATTTTGTTCCTTGTGTATCA	952	51	
SD_SA1_c1_14147N	GGTTTAGGATAGAAACCAACCTG			
SD_SA1_c2_832J	TGAATTACTCAAAACCCGACAA	309	53	
SD_SA1_c2_174N	GCAGATTGAAAAATAACAACAATTC			
SD_SA1_c2_472J	TTTCTTTTAAATTCTCCTTTTTCAAGA	238	54	
SD_SA1_c2_664N	GCGCGTGGTTTAACAAAGAT			
SD_SA1_c2_692J	AAACCACGCGCGTATAAAAC	209	49	
SD_SA1_c2_862N	AATTTTGCCTTAATTTGCTT			
SD_SA1_c1_12378J	TTTCCCCAGCCTAAAATAATG	853	48	Lane 1
SD_SA1_c2_141N	AGTGGGTGATTAGAAAATATAATAA			
SD_SA1_c2_165J	TTATTATATTTCTAATCACCCACT	405	48	Lane 2
SD_SA1_c2_521N	TTTCTGTTATAAAAACAACAAAGAT			
SD_SA1_c2_545J	ATCTTTGTTGTTTTTATAACAGAAA	324	48	Lane 3
SD_SA1_c1_13302N	TTAACAATTATGAAACATTGAACAC			
SD_SA1_c2_545J	ATCTTTGTTGTTTTTATAACAGAAA	566	48	Lane 4
SD_SA1_c2_141N	AGTGGGTGATTAGAAAATATAATAA			
SD_SA1_c1_12940J	CAAGCCTCCAAAAGCAAAAG	291	47	Additional PCR experiments: no product and no evidence for coexisting unipartite and bipartite genomes in SA1
SD_SA1_c2_141N	AGTGGGTGATTAGAAAATATAATAA			
SD_SA1_c1_12378J	TTTCCCCAGCCTAAAATAATG	886	49	
SD_SA1_c2_174N	GCAGATTGAAAAATAACAACAATTC			
SD_SA1_c1_12940J	CAAGCCTCCAAAAGCAAAAG	671	47	
SD_SA1_c2_521N	TTTCTGTTATAAAAACAACAAAGAT			
SD_SA1_c2_165J	TTATTATATTTCTAATCACCCACT	621	47	
SD_SA1_c1_13219N	TCATGATACACAAGGAACAAAA			
SD_SA1_c2_472J	TTTCTTTTAAATTCTCCTTTTTCAAGA	314	49	
SD_SA1_c1_13219N	TCATGATACACAAGGAACAAAA			
SD_SA1_c2_165J	TTATTATATTTCTAATCACCCACT	704	47	
SD_SA1_c1_13302N	TTAACAATTATGAAACATTGAACAC			

[a]Primer naming convention: species complex_cryptic species_chromosome (cryptic species SA1 only)_location in genome and J (majority coding) or N (minority coding) strand

[b]Note corresponds to experiments validating the bipartite genome architecture of SA1 (figure 1)

Additional file continued: Capillary sequencing primers, estimated fragment size, and annealing temperature (T_A) used to validate next generation sequenced Scirtothrips dorsalis mitochondrial genomes.

Primer[a]	Sequence	Size	T_A
SD_EA1_15123J	GCTACCTCGATGTTGAATTAAGG	853	54
SD_EA1_590N	CCCCTGCAAGAACAGGTAAAG		
SD_EA1_95J	TCAGGAATACTGGGGCTGTC	913	54
SD_EA1_967N	TGGAACCACAAAATGTAGCAAG		
SD_EA1_761J	TCCAGGGTTCGGATTAATTTC	927	54
SD_EA1_1646N	TCCACATTCAAAGGGTCTCC		
SD_EA1_1328J	CACGACGATACTCTGACTACCC	985	54
SD_EA1_2269N	CAATATCATTGAGCACCAAAGAC		
SD_EA1_2267J	AAATCCCAGGGTTACTACAAA	491	55
SD_EA1_2715N	GGTCGAAACTAAGAACAGAAGCA		
SD_EA1_2606J	AATTTGTGGAACAGGACATGC	995	54
SD_EA1_3557N	ATGGCCTTGGGGATACTAGG		
SD_EA1_3275J	GAAATTCTAATCCTGATCCTCTGG	996	54
SD_EA1_4227N	GCTGCTTCAAAGCCAAAATG		
SD_EA1_4120J	GCAGATTCAGTCTATGGGTCAAC	990	54
SD_EA1_5066N	CCCCATTAGCATGTAAAATTCG		
SD_EA1_5682J	GCGATCCTTCGGTCTATTCC	724	54
SD_EA1_6361N	TCAATAACTGAAATAACTCAAAATTGG		
SD_EA1_6111J	GTTTTCCTGGGGCTTTCATC	938	54
SD_EA1_7004N	TCTCATAGAATTAGGGCTATTGG		
SD_EA1_7208J	TGCAATTACGATTAGGTCCAA	508	55
SD_EA1_7676N	GCGAAATCAAAGGGAGTACG		
SD_EA1_7699J	CTCCCTTTGATTTCGCAGAG	681	59
SD_EA1_8341N	TTTAAGTGTGACCGCGACTG		
SD_EA1_8259J	TTTAAAACATGGAATATTAAGCAAG	991	54
SD_EA1_9205N	TCGAGATTCGGGTAGGTTTG		
SD_EA1_8874J	ACAAATTGCCCGTCACTCTC	916	54
SD_EA1_9752N	TTGGCGTAGTCTGATTCGTC		
SD_EA1_10161J	TTGCTGATTCCCTTTAAACCTC	968	54
SD_EA1_11085N	GCTCCAACCCCTGTATCTTC		
SD_EA1_10833J	AGCCTTAAACCCAGCATGAG	954	54
SD_EA1_11743N	AAAACGTGGACTTGTGGAGTC		
SD_EA1_11572J	GAGAAGAAATATAATCCAAATATAAAGG	951	54
SD_EA1_12622N	AGCTCATGTTGAGGCTCCTG		
SD_EA1_12485J	CGAATGCAAATCAATCTTGTC	880	54
SD_EA1_13322N	TTGTTTCTGAGAGGGCAGTG		
SD_EA1_13713J	TCATCTTTTCTAACCACCCTTT	434	50
SD_EA1_14101N	TTTTTATTTTGTTGACCTCCTTTTT		
SD_EA1_14384J	TTTTCTTTGTGTTTAATGTTTCAC	868	54
SD_EA1_15207N	GGTTTAGGATAGAAACCAACCTG		

[a]Primer naming convention: species complex_cryptic species_chromosome (cryptic species SA1 only)_location in genome and J (majority coding) or N (minority coding) strand
